# Effects of vorticity on solitary waves

**DOI:** 10.1038/s41598-022-23267-w

**Published:** 2022-11-02

**Authors:** Keisuke Nakayama, Kojiro Tani, Hideto Yoshimura, Ichiro Fujita

**Affiliations:** 1 Graduate School of Engineering, Kobe University, Kobe, 657-8501 Japan; 2 Mizuho Research & Technologies, Ltd., Tokyo, Japan; 3 Construction Engineering Research Institute, Kobe University, Kobe, Japan

**Keywords:** Civil engineering, Physical oceanography

## Abstract

The vorticity effect on solitary wave profiles has not been solved experimentally; previous studies theoretically and numerically showed that when a solitary wave progressed in the positive direction, the effective wavelength of a solitary wave with positive vorticity increased. Using laboratory experiments and fully nonlinear numerical simulations, we here show that the effective wavelength is extended more when positive vorticity is given to a progressive wave in the positive direction. We further show that the total energy increases with increasing positive vorticity, demonstrating that a wave with positive vorticity propagates with less attenuation and lasts longer than a solitary wave with no vorticity. We anticipate that our outcomes will provide a starting point for more sophisticated methods to investigate the effect of vorticity on solitary waves in laboratory experiments and numerical simulations.

## Introduction

When surface waves progress over a sandy or rough bottom, the bottom friction induces vorticity, which affects the characteristics of surface waves, such as the vertical profile of the horizontal velocity and the wave shape^1^. Mean currents have similar effects: positive and negative vorticities correspond to the surface wave propagating opposite to the direction of and in the same direction of current. Dean showed the possibility that mean currents deform the shapes of surface waves^2^. Later studies revealed the importance of current effects on the surface wave amplitudes in a wave–current system by considering the logarithmic vertical profile of horizontal velocities^3,4^.

On the other hand, other studies demonstrated the importance of vorticity on the surface wave profiles. For example, Freeman and Johnson^5^ derived the Korteweg and de Vries equation in shear flows, arriving at the same result as Benjamin^6^ by following the original strategy of Burns^7^. They mentioned the necessity for numerical computation to evaluate detailed velocity profiles and wave speeds. Dalrymple^8^ presented a finite amplitude wave in a constant shear flow using a numerical perturbation procedure, which led to a change in wavelength propagating over a vertically varying linear shear current; still, he mentioned the necessity of laboratory experiments to verify his results. Teles and Peregrine^9^ showed that waves with more extended wavelength occurred when positive vorticity was given to a progressive wave in the positive direction, corresponding to surface waves that progress against a stream in an open channel flow. Vanden-Broeck^10^ investigated a finite amplitude wave in constant vorticity by using a boundary integral equation method. In addition, the effect of continuous and discontinuous vorticities on surface waves has been shown^11–14^. For example, a fundamental investigation showed that, when surface waves progress in the positive direction, wave amplitude increases as vorticity increases from negative to positive before the waves break^11^. In the same study, the authors performed a detailed analysis of the shear-layer effect on wave shape.

Teles and Peregrine^9^ suggested the need to consider “strong nonlinearity” when analyzing the propagation of steady surface waves with constant vorticity. Through strongly nonlinear long gravity wave equations, Choi^15^ demonstrated that the wavelength of a solitary wave increased under positive vorticity and decreased under negative vorticity, which was similar to the findings of Teles and Peregrine^9^. Moreira and Peregrine^16^ modelled the underlying current as a distribution of singularities (vortices) using a boundary integral method. Lannes and Marche^17^ developed extended Green–Naghdi equations with additional advection-like equations for the vorticity-related terms and investigated solitary waves numerically. As a fully nonlinear model, a direct numerical method has been proposed to simulate nonlinear surface waves with nonzero constant vorticity^18^. However, in contrast to the various numerical simulations, there have been few laboratory experiments investigating the effect of vorticity on the amplitude and wavelength of a solitary wave. Therefore, it is necessary to conduct laboratory experiments and develop a method including full nonlinearity that enables verification of the deformation of surface waves under a constant shear flow.

In previous studies on one-layer models, which can include “strong nonlinearity” of waves, the Boussinesq-type equations^19–21^ have been improved to include strong nonlinear waves^22–25^. In contrast to a one-layer model, studies examining strong nonlinear waves in a two-layer system indicated the possibility of large-amplitude internal solitary waves due to the effect of topography and the excitation of internal solitary waves^26,27^. Two-layer systems with constant vorticity in each fluid layer have been proposed to investigate nonlinear responses generated by vorticity^28^. In a theoretical study on a perturbation basis, Choi and Camassa^29^ derived two sets of internal-wave equations by considering the full nonlinearity of internal waves in a two-layer system, where one set of equations was chosen to treat a shallow layer whether it lay on another shallow layer with weak dispersion or on a deep layer with intermediate dispersivity^11^. The models mentioned above can include strong nonlinearity, but they are based on nonlinear long waves. Therefore, it may be necessary to apply fully nonlinear wave equations to investigate the vorticity effect on solitary waves, as in the study by Guyenne^18^.

Nakayama and Kakinuma^30^ proposed the Fully nonlinear and strongly Dispersive Internal wave equations in a two-layer system (FDI-2s equations) based on Isobe^31^ and Luke^32^. The collective model name for the fully nonlinear and strongly dispersive wave equations is the Nakayama model^33–35^. Although the Nakayama model analyzes internal waves in a multi-layer system, it may be possible to analyze surface water waves considering that the upper and lower layers are air and water, respectively. In addition, it is easy to include vorticity effects in the Nakayama model because of the use of the variational principle^36,37^; Luke first suggested the possibility of applying the variational principle to vorticity flow fields. Therefore, we attempted to expand the Nakayama model to include new equations that can include vorticity effects. The effect of vorticity on solitary waves was numerically investigated to determine whether waves with more extended wavelength, which correspond to surface waves that progress against a stream in an open channel flow, could be reproduced. Moreover, we conducted rough-bottom laboratory experiments to investigate the effect of negative vorticity on a progressive solitary wave in the positive direction.

### Laboratory experiments

**Figure 1:**
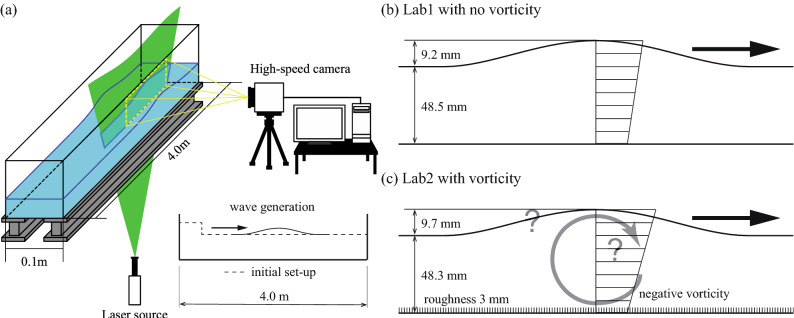
Laboratory experiments. (**a**) Schematic diagram of the laboratory experiments. A laser sheet was inserted through the bottom slit. (**b**) Lab1 with no vorticity. (**c**) Lab2 with vorticity.

Many previous results have been obtained using theoretical analyses and numerical simulations, but their have been an insufficient number of analyses using laboratory experiments. Therefore, we attempted to conduct laboratory experiments to investigate the vorticity effect on solitary waves by using 3 mm roughness (TOWA: DMAH-9211) at the bottom of an open channel (Fig. 1). This roughness drives negative vorticity on a progressive solitary wave in the positive direction due to the friction effect from the bottom. Two cases were tested: a smooth-bottom case (Lab1, with no vorticity) and a rough-bottom case (Lab2, with vorticity) (Table 1). We tried to produce a similar wave height in both cases, 9.2 mm and 9.7 mm for Lab1 and Lab2, respectively. The wave was generated by exerting a step-like shape displacement to create one solitary wave. Particle Image Velocimetry (PIV) was applied to measure the vertical profiles of horizontal velocities and the wave shapes Lab1 and Lab2 (Fig. 1). The width and length of the acrylic open channel were 0.1 m and 4.0 m. Since the both-ends vertical walls of the open channel reflect waves perfectly, we conducted our measurements after the waves had been reflected twice from the vertical wall, meaning the effective open channel length was 12 m and long enough to reproduce a solitary wave. Since the laboratory apparatus was too small to allow sufficient alterations of the total water depth and wave height, we examined only two conditions with negative vorticity. Note that our laboratory experiment could not give positive vorticity to progressive surface waves in the positive direction, so we used numerical simulations to investigate the positive vorticity effect on solitary waves.

**Table 1:** Conditions for solitary wave cases.

Case	${\eta }_{\mathrm{max}}-{h}_{2}$ (m)	${\omega }_{0}$ (s^−1^)	${F}_{V}={\omega }_{0}{h}_{2}/\sqrt{{gh}_{2}}$	${\lambda }_{I}$ (m)
Lab1	0.0092	0	0	0.273
Lab2	0.0100	− 0.114	− 0.0077	0.262
A1	0.207	0.0	0	14.76
A2	0.204	0.25	0.113	15.64
A3	0.202	0.50	0.226	17.12
A4	0.210	− 0.25	− 0.113	13.72
A5	0.214	− 0.50	− 0.226	12.34
B1	0.37	0	0	11.15
B2	0.36	0.25	0.113	12.44
B3	0.34	0.50	0.226	14.09
B4	0.39	− 0.25	− 0.113	9.99
B5	0.40	− 0.50	− 0.226	7.93
C1	0.79	0.0	0	8.00
C2	0.73	0.25	0.113	8.83
C3	0.66	0.50	0.226	10.08
C4	0.84	− 0.25	− 0.113	6.96
C5	0.89	− 0.50	− 0.226	5.48

${\eta }_{\mathrm{max}}$ is the maximum water depth from the bottom, ${h}_{2}$ is the initial water depth, ${\omega }_{0}$ is the vorticity, and ${\lambda }_{I}$ is the effective wavelength.

## Results and discussion

### Laboratory experiments

**Figure 2:**
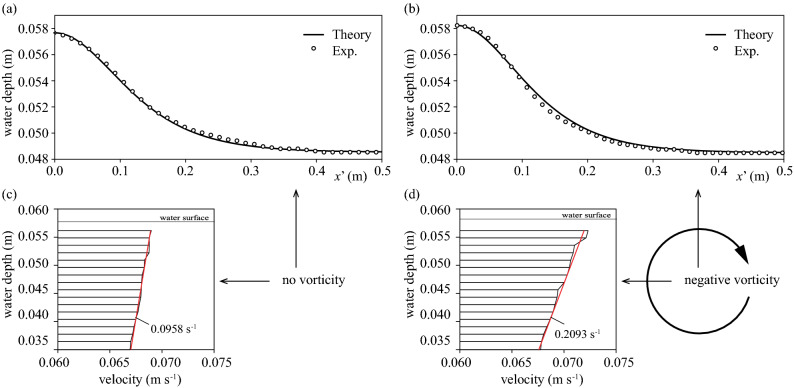
Wave profile and horizontal velocity. (**a**) Wave profile of Lab1 with no vorticity. (**b**) Wave profile of Lab2 with vorticity. Solid lines indicate the Fenton’s solution, and circles indicate the water depth by laboratory experiments. (**c**) Vertical profile of the horizontal velocity at the crest of Lab1 with no vorticity. (**d**) Vertical profile of the horizontal velocity of Lab2 with vorticity.

The water depths and wave heights were 0.0485 m and 0.0092 m in Lab1, with no vorticity, and 0.0484 m and 0.0097 m in Lab2, with vorticity. The wave celerity of Lab1 was 0.689 m s^−1^, which was smaller than the theoretical solution of 0.754 m s^−1^. Acrylic was used in the laboratory experiment; still, the friction from the bottom and lateral walls was expected to reduce the wave celerity. The wave celerity of Lab2 was 0.59 m s^−1^, smaller than that of Lab1 due to the additional bottom friction. The wave shape of Lab1 agreed very well with that by Fenton's solution^39^ (Fig. 2a). In contrast, Fenton's solution overestimated the wave shape of Lab2 in the trough area (Fig. 2b). It was demonstrated that Fenton’s solution agreed very well with the fully nonlinear and strongly dispersive surface wave^26^, suggesting that the surface wave of Lab1 was a typical solitary wave. Choi^11^ demonstrated that the water depth in the trough area decreases by giving negative vorticity to the progressive solitary wave in the positive direction, which agreed with our laboratory experiments.

The influence of vorticity on solitary waves was evaluated using the Froude number (${F}_{V}$) defined by Choi^11^ as the ratio of vorticity × water depth and wave celerity. The vertical profile of horizontal velocities obtained from the PIV analysis showed the typical velocity profile of a solitary wave in Lab1 (Fig. 2c). The vertical profile of horizontal velocities was linear from the bottom to the solitary wave height. In contrast, the vertical gradient of horizontal velocities in Lab2 was 0.2093 s^−1^, greater than the 0.0958 s^−1^ of Lab1 (Fig. 2d), suggesting that the bottom roughness induced negative vorticity. Note that the 0.0958 s^−1^ of Lab1 is not a vorticity, but the vertical profile of horizontal velocities due to the typical characteristic of a solitary wave. The vertical gradient of horizontal velocities was obtained using the least-squares method, which gave the Froude number, ${F}_{V}=-0.0077$ (red lines in Fig. 2d). If we assume the celerity to be a linear longwave celerity, the Froude number is ${\omega }_{0}\sqrt{{h}_{2}}/\sqrt{g}$ (${\omega }_{0}$: vorticity; ${h}_{2}$: water depth; $g$: gravity acceleration). Thus, as the Froude number is proportional to the square root of water depth when vorticity is constant, the Froude number tends to be smaller in a shallow water depth laboratory experiment than in the results of previous studies.

To confirm the solitary-wave deformation due to the vorticity effect, we computed the effective wavelengths, ${\lambda }_{I}$, by following the definition used by Koop and Butler^38^:

$${\lambda }_{I}=\frac{1}{{a}_{H}}{\int }_{-\infty }^{\infty }\left(\eta -{h}_{2}\right)dx$$

where ${\lambda }_{I}$ is the effective wavelength, and *a*_*H*_ is the wave height.

In Lab1 and Lab2, the effective wavelength of Lab2, 0.265 m, was shorter than that of Lab1, 0.273 m, which may be due to the negative vorticity effect (Table 1). The previous studies demonstrated the possibility of both longer and shorter effective wavelengths when negative vorticity was given. Thus, the fact that the negative vorticity shortens the effective wavelength may provide a scientifically significant outcome similar to that in Choi^11^; still, the difference was too small to confirm the deformation of a solitary wave because of the small Froude number, ${F}_{V}= -0.0077$, which is substantially smaller than that of Choi^11^. In the laboratory experiment, the maximum vorticity is expected to be 0.25 s^−1^, even though we use robust roughness. In addition, the maximum water depth may be 0.5 m, even using a long wave tank, which provides a Froude number of 0.056 using a linear longwave speed. In a natural setting, the maximum vorticity may be twice as much as in the laboratory experiment. The water depth is up to a few meters, providing a Froude number of 0.28, more than five times the Froude number in the laboratory experiment. Since the size of the Froude number limits laboratory experiments, it is necessary to apply numerical simulations to analyze more larger Froude numbers corresponding to those produced by actual phenomena. Indeed, the Froude number in our laboratory experiments was too small to capture the actual characteristics of the constant vorticity effect on the amplitude and wavelength of a solitary wave. Therefore, in numerical simulations, the water depth was 40 times greater than in the laboratory experiments so as to obtain a more significantly larger Froude number. Note that the numerical simulation cannot estimate the vorticity induced by the bottom friction in the laboratory experiment. Thus, we cannot make direct comparisons between the laboratory experiments and the numerical simulation.

### Numerical simulations

**Figure 3:**
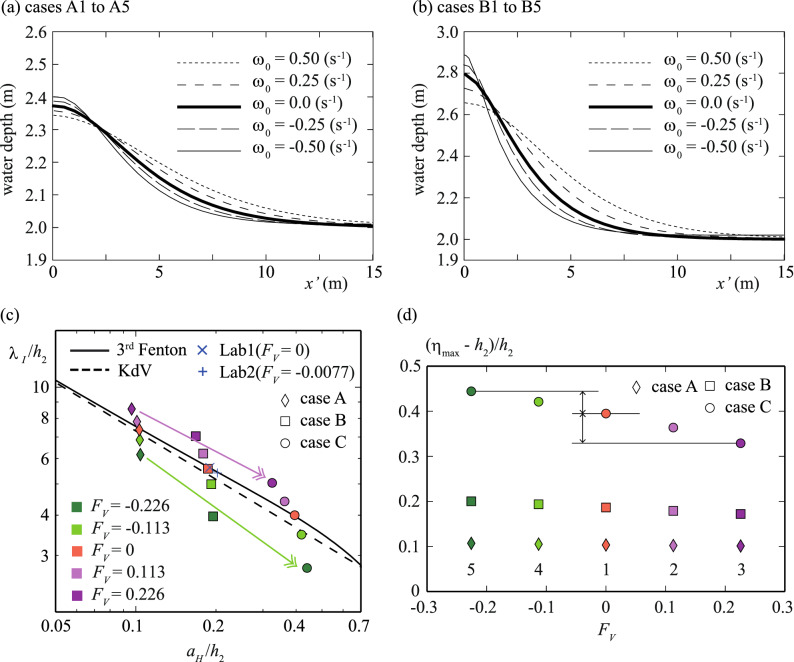
Comparisons of water depth distributions at 20 s. (**a**) Dotted, broken spaced, thick solid, broken, and thin solid lines indicate cases B3, B2, B1, B4, and B5, respectively. (**b**) Dotted, broken spaced, thick solid, broken, and thin solid lines indicate cases C3, C2, C1, C4 and C5, respectively. (**c**) ${\lambda }_{I}$ for theoretical solutions and the Nakayama model at 20 s. Broken and solid lines indicate the KdV solution and the 3rd-order solution by Fenton^39^ for surface waves. (**d**) Froude number, ${F}_{V}$, and the normalized maximum wave height, $\left({\eta }_{\mathrm{max}}-{h}_{2}\right)/{h}_{2}$, for cases A, B, and C at 20 s.

We show results using the Nakayama model for cases B and C only, as these cases exhibit more obvious differences induced by the vorticity effect than case A, where the wave height is the smallest (Table 1). We will use case A to discuss the vorticity effect on wave height and wavelength. The values of the horizontal velocity component at the crest, ${U}_{S}$ ($={\omega }_{0}\left({h}_{2}+{\eta }_{max}\right)$) m s^−1^, due to the vorticity for ${\omega }_{0}=0.0$ s^−1^, ${\omega }_{0}=0.25$ s^−1^, ${\omega }_{0}=0.50$ s^−1^, ${\omega }_{0}=-0.25$ s^−1^, and ${\omega }_{0}=-0.50$ s^−1^, were obtained as (${U}_{S}=$) 0.0 m s^−1^, 0.59 m s^−1^, 1.17 m s^−1^, − 0.60 m s^−1^, and − 1.20 m s^−1^ in cases B1 to B5, respectively. The celerity of a solitary wave with no vorticity, *C*_*R*_ m s^−1^, was obtained as 4.89 m s^−1^, and the values of the Froude number, ${\omega }_{0}{h}_{2}/\sqrt{g{h}_{2}}$, were 0.0, − 0.113, − 0.226, 0.113, and 0.226 in cases B1 to B5. In cases B1 to B5, ${\lambda }_{I}$ in the case with ${\omega }_{0}=0.50$ s^−1^ was the longest (Fig. 3a and Table 1). Note that a positive Froude number corresponds to a case where a solitary wave progresses against a stream.

In cases C1 to C5, where wave amplitude was largest, the values of the horizontal velocity components due to the vorticity at the crest, *U*_*S*_ m s^−1^, at the maximum wave height were 0.0 m s^−1^, 0.68 m s^−1^, 1.33 m s^−1^, − 0.71 m s^−1^, and − 1.45 m s^−1^ (Table 1). The wave celerity with no vorticity was *C*_*R*_ = 5.55 m s^−1^, and the values of the Froude number, ${\omega }_{0}{h}_{2}/\sqrt{g{h}_{2}}$, were 0.0, − 0.113, − 0.226, 0.113, and 0.226 from cases C1 to C5. In cases C1 to C5, ${\lambda }_{I}$ in the case with ${\omega }_{0}=0.50$ s^−1^ was the longest (Fig. 3b and Table 1).

### Vorticity effect on wavelength, amplitude and energy

Laboratory experiments demonstrated that the wave height decreased around the skirt of a solitary wave when the wave progressed positively with negative vorticity, as in Choi^11^. The laboratory experiment results of Lab1, with no vorticity, showed that the relationship between the wave height, ${a}_{H}$, and effective wavelength agreed with Fenton's theoretical solution^39^ (Fig. 3c). In contrast, the effective wavelength of Lab2, with negative vorticity, was slightly smaller than the theoretical solution. However, the difference in the effective wavelength was tiny, as the Froude number was − 0.0077; this was too small to confirm the deformation of a solitary wave.

Therefore, the Nakayama model was applied using a water depth of 2.0 m, which was 40 times greater than in the laboratory experiment. The effective wavelengths were confirmed to agree with Fenton's solutions when there was no vorticity (Fig. 3c). To investigate the change in effective wavelength according to wave height, the small-amplitude case A was added in Fig. 3c. In the maximum positive vorticity cases B3 and C3, the effective wavelengths were 1.41 and 1.46 times greater than in the no-vorticity cases B1 and C1, as in the previous study^40^. In contrast, the effective wavelengths of the minimum negative vorticity cases B5 and C5 were 0.71 and 0.69 times smaller than in the no-vorticity cases. The ratio of the effective wavelength based on the no-vorticity condition was almost constant when vorticity was constant (purple arrows in Fig. 3c). However, the ratio of the negative vorticity cases decreased as wave height increased (green arrows in Fig. 3c). Therefore, these experiments may have uncovered a new scientific relation—namely, the higher the wave height, the greater the effect of negative vorticity on the effective wavelength of a solitary wave shape compared to that of positive vorticity.

On the other hand, the maximum wave height defined as $\left({\eta }_{\mathrm{max}}-{h}_{2}\right)/{h}_{2}$ increased as the Froude number, ${F}_{V}$, decreased; the more significant the initial wave height, the greater the change in wave height (Fig. 3d). In case C, where the initial wave height is the maximum, the change in the maximum wave height of the positive vorticity case was more significant than that of the negative vorticity case (arrows in Fig. 3d). Note that the Froude number showed no significant influence on the maximum wave height when the normalized initial wave height was less than 0.1.

To understand the energetics of a solitary wave affected by vorticity, the normalized total energy was computed by using the wave amplitude and wavelength obtained from the KdV theory:

$$\begin{aligned} {E}_{T}&=\frac{1}{{a}_{H}^{2}{\lambda }_{KdV}g}{\int }_{-\infty }^{\infty }\left\{\frac{g}{2}{\left(\eta -{h}_{2}\right)}^{2}+{\int }_{b\left(x\right)}^{\eta }\frac{1}{2}\left({u}^{2}+{w}^{2}\right)dz\right\}dx \\ & =\frac{1}{2g}\sqrt{\frac{3}{{a}_{H}^{3}{h}_{2}^{3}}}{\int }_{-\infty }^{\infty }\left\{\frac{g}{2}{\left(\eta -{h}_{2}\right)}^{2}+{\int }_{b\left(x\right)}^{\eta }\frac{1}{2}\left({u}^{2}+{w}^{2}\right)dz\right\}dx \end{aligned}$$

where ${E}_{T}$ is the normalized total energy and ${\lambda }_{KdV}$ is the KdV wavelength.

**Figure 4:**
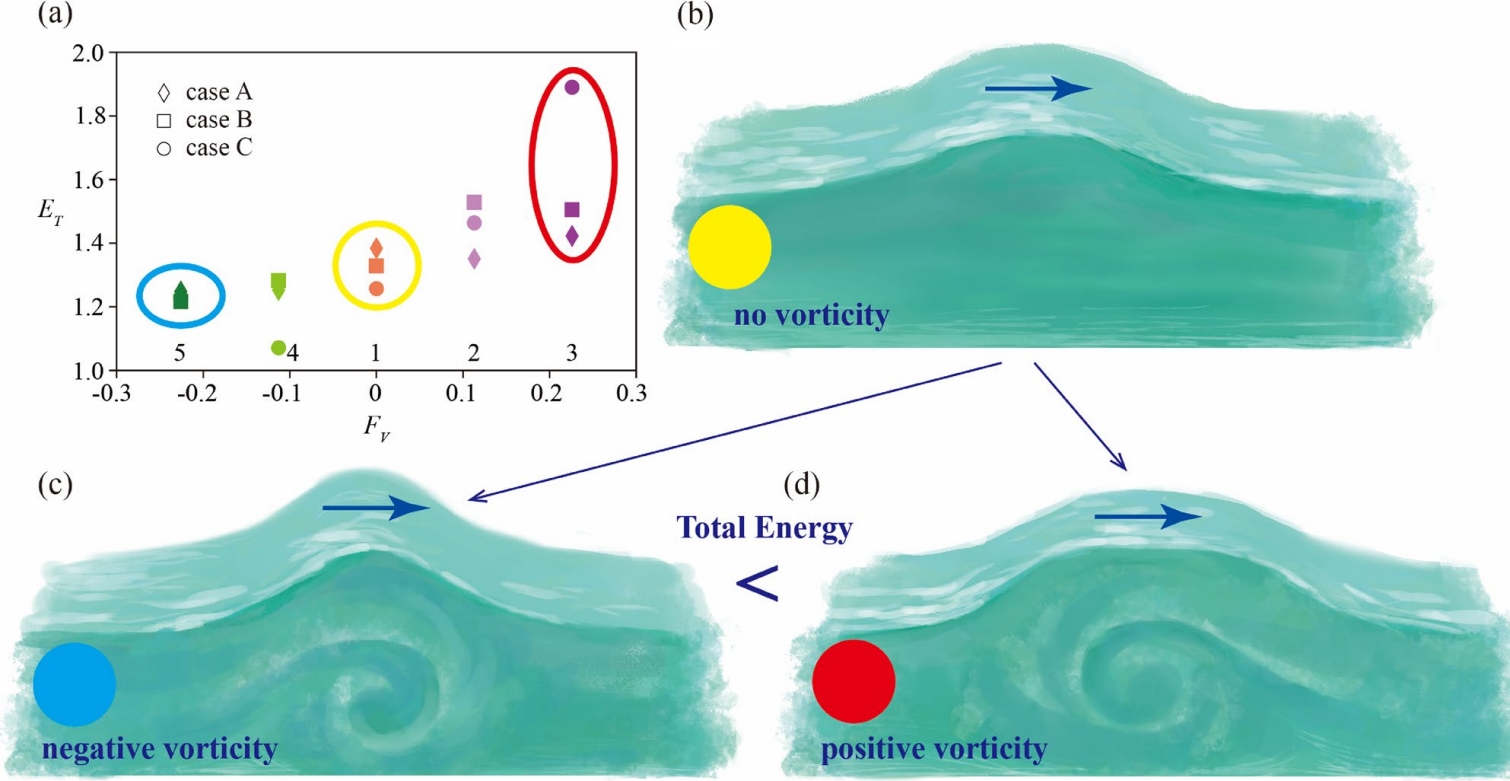
Total energy and vorticity. (**a**) Froude number, ${F}_{V}$, and the normalized total energy, ${E}_{T}$, for cases A, B, and C at 20 s. (**b**) Solitary wave profile of no vorticity. (**c**) Solitary wave profile of negative vorticity. **d**) Solitary wave profile of positive vorticity. Illustration adapted with permission from Reina Nakayama.

It was shown that positive vorticity tends to have greater total energy (Fig. 4a). Since a wave with positive vorticity had a longer effective wavelength, as shown in Fig. 3c, the normalized total energy was greater in a wave with positive vorticity (Fig. 4). Significantly, the larger the vorticity, the more significant the normalized total energy. These results may suggest that a wave with positive vorticity propagates with less attenuation and lasts longer than a solitary wave with no vorticity.

## Methods

### Laboratory experiments

The wave shape and velocity vectors were visualized by sliding a laser sheet (Japan Laser DPGL-2w) with a thickness of 0.005 m through the slit at the open channel bottom and using crushed nylon particles with a representative scale of 80 µm. Wave shape was measured using a high-speed camera, and the velocities were obtained using PIV analysis^41^. The camera was a CASIO EX100 with a sampling rate of 30 frames per second and a resolution of 3840 × 2160. An area of 0.05 m adjacent to the water surface was measured to obtain high-resolution images with a grid size of 0.00005 m. A solitary wave was generated by releasing a steplike convex region dammed up by the acrylic plate at the upstream end. The Lab2 wave shape with vorticity was generated with a staggered arrangement projection of 1 mm length. The laser sheet was slid through a slit, measuring 0.01 m wide and 0.3 m long, at the open channel bottom. Before generating a solitary wave, we confirmed the absence of any vorticity or turbulence in an open channel with particles for the PIV. We also carried out laboratory experiments repeatedly to obtain similar wave heights in both Lab1 and Lab2 to the greatest extent possible.

### Numerical simulations

**Figure 5:**
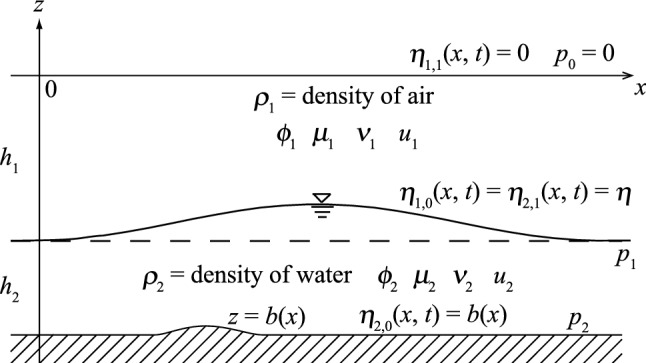
A two-layer fluid for water waves.

We attempted to use the Nakayama model to investigate the effect of vorticity on a solitary wave. In applying the variational principle, the functional including the vorticity effect by Luke^32^ yields the Euler‒Lagrange equations by using velocity potential^31^. The Euler‒Lagrange equations were solved numerically based on the numerical scheme developed by Nakayama and Kakinuma^30^. They assumed wave fields for the irrotational case, but in the present study, the rotational condition including vorticity effects was introduced in a two-layer fluid by following Clebsch^36^, Bateman^37^, and Luke^32^ ((1) and Fig. 5). The second term of (1) indicates the velocity potential due to the vorticity effect. Details are shown below.

1 $${{\varvec{u}}}_{l}=\left({u}_{l},{v}_{l}\right)=\nabla {\phi }_{l}+{\mu }_{l}\nabla {\nu }_{l}\,\, \mathrm{and}\,\, {w}_{l}=\frac{\partial {\phi }_{l}}{\partial z}+{\mu }_{l}\frac{\partial {v}_{l}}{\partial z}$$

Here, ${u}_{l}$ is the velocity of the *l*th layer in the *x* direction, ${v}_{l}$ is the velocity of the *l*th layer in the *y* direction, ${\mu }_{l}$ and ${\nu }_{l}$ are the vorticity effect terms, ${\phi }_{l}$ is the velocity potential, ${w}_{l}$ is the vertical velocity of the *l*th layer, and $\nabla$ is a partial differential operator in the horizontal plane, i.e., =  $(\partial / \partial x, \partial / \partial y)$.

The upper and lower layers are represented as $l$ = 1 and 2 in a two-layer system, respectively, and incompressible fluids are assumed to be stable in still water, as shown in Fig. 5. We consider that the upper and lower layers are the air and water, respectively. The friction term between the air and water was ignored in the numerical simulations. The *l*th layer thickness in still water is denoted by ${h}_{l}$, and two-layer fluids will not mix during wave motion. The density is spatially uniform and temporally constant in each layer. In the *l*th layer, the upper and lower interfaces are indicated as ${\eta }_{l,1}$ and ${\eta }_{l,0}$, respectively, and the pressure on the lower interface is *p*_*l*_. By following Isobe^31^ based on Clebsch^36^ and Luke^32^, the function for the variational problem in the *l*th layer, ${F}_{l}$, was determined by

2 $${F}_{l}\left[{\phi }_{l},{\mu }_{l},{\nu }_{l},{\eta }_{i,j}\right]={\int }_{{t}_{0}}^{{t}_{1}}{\iint }_{A}{\int }_{{\eta }_{l,0}}^{{\eta }_{l,1}}\left\{\frac{\partial {\phi }_{l}}{\partial t}+{\mu }_{l}\frac{\partial {v}_{l}}{\partial t}+\frac{1}{2}{\left(\nabla {\phi }_{l}+{\mu }_{l}\nabla {\nu }_{l}\right)}^{2}\right.\left.+\frac{1}{2}{\left(\frac{\partial {\phi }_{l}}{\partial z}+{\mu }_{l}\frac{\partial {\nu }_{l}}{\partial z}\right)}^{2}+gz+\frac{{p}_{l-j}+{P}_{l}}{{\rho }_{l}}\right\}dzdAdt$$

3 $${P}_{l}=\sum \limits_{k=1}^{l-1}\left({\rho }_{l}-{\rho }_{k}\right)g{h}_{k}$$

where $g$ is gravitational acceleration; plane *A*, which is the orthogonal projection of the object domain onto the $x{-}z$ plane, is assumed to be independent of time.

To derive a set of equations, vertical integration was performed analytically by expanding ${\phi }_{l}$, ${\mu }_{l}$, and ${\nu }_{l}$. ${\phi }_{l}$, ${\mu }_{l}$, and ${\nu }_{l}$ were expanded into a series in terms of $\alpha$ given a set of vertically distributed functions, ${Z}_{l,\alpha }$, ${M}_{l,\alpha }$, and ${L}_{l,\alpha }$, multiplied by their weightings, ${f}_{l,\alpha }$, ${m}_{l,\alpha }$, and ${n}_{l,\alpha }$, respectively.

4 $${\phi }_{l}\left(x,z,t\right)=\sum \limits_{\alpha =0}^{N-1}\left\{{Z}_{l,\alpha }\left(z\right){f}_{l,\alpha }\left(x,t\right)\right\}\equiv {Z}_{l,\alpha }{f}_{l,\alpha }$$

5 $${\mu }_{l}\left(x,z,t\right)={\sum }_{\alpha =0}^{N-1}\left\{{M}_{l,\alpha }\left(z\right){m}_{l,\alpha }\left(x,t\right)\right\}\equiv {M}_{l,\alpha }{m}_{l,\alpha }$$

6 $${\nu }_{l}\left(x,z,t\right)=\sum \limits_{\alpha =0}^{N-1}\left\{{L}_{l,\alpha }\left(z\right){n}_{l,\alpha }\left(x,t\right)\right\}\equiv {L}_{l,\alpha }{n}_{l,\alpha }$$

Here, $N$ is the total number of an expanded functions.

(7) is applied to Eqs. (4) to (6).

7 $${Z}_{l,\alpha }={M}_{l,\alpha }={L}_{l,\alpha }={z}^{\alpha } \left(\alpha =\mathrm{0,1},2, \ldots ,N-1\right)$$

We substituted (3)–(6) into (2), after which the function was integrated vertically. Then we applied the variational principle to obtain the following Euler–Lagrange equations for each layer, i.e., the equations for fully nonlinear and strongly dispersive internal waves with vorticity effects. We call the fully nonlinear and strongly dispersive internal wave equations with vorticity effects in a two-layer system the “FDV-2s” equations. The collective model name for the FDS, FDI-2s, FDI-3s, FDV-2s and FDI-multi-layer-system equations is the Nakayama model.

[1st layer]

In the 1st layer, i.e., the upper layer, *l* = 1 and *j* = 0, and the Euler − Lagrange equations become

8 $$-{\eta }^{\alpha }\frac{\partial \eta }{\partial t}+\nabla \left({Q}_{1}\left[\alpha +\beta \right]\nabla {f}_{1,\beta }\right)-{R}_{1}\left[\alpha ,\beta ,0\right]{f}_{1,\beta }+\nabla \left({Q}_{1}\left[\alpha +\beta +\gamma \right]{m}_{1,\beta }\nabla {n}_{1,\gamma }\right)-{R}_{1}\left[\alpha ,\gamma ,\beta \right]{m}_{1,\beta }{m}_{1,\gamma }=0$$

9 $${Q}_{1}\left[\alpha +\beta \right]\frac{\partial {m}_{1,\beta }}{\partial t}-{\eta }^{\alpha +\beta }{m}_{1,\beta }\frac{\partial \eta }{\partial t}+\nabla \left({Q}_{1}\left[\alpha +\beta +\gamma \right]\nabla {f}_{1,\beta }{m}_{1,\gamma }\right)-{R}_{1}\left[\alpha ,\gamma ,\beta \right]{f}_{1,\beta }{m}_{1,\gamma }+\nabla \left({Q}_{1}\left[\alpha +\beta +\gamma +\delta \right]{m}_{1,\beta }{m}_{1,\gamma }\nabla {n}_{1,\delta }\right)-{R}_{1}\left[\alpha ,\gamma ,\beta +\delta \right]{m}_{1,\beta }{m}_{1,\gamma }{n}_{1,\delta }=0$$

10 $${Q}_{1}\left[\alpha +\beta \right]\frac{\partial {n}_{1,\beta }}{\partial t}+{Q}_{1}\left[\alpha +\beta +\gamma \right]\nabla {f}_{1,\beta }{\nabla n}_{1,\gamma }+{R}_{1}\left[\beta ,\gamma ,\alpha \right]{f}_{1,\beta }{n}_{1,\gamma }+{Q}_{1}\left[\alpha +\beta +\gamma +\delta \right]{m}_{1,\beta }\nabla {n}_{1,\gamma }\nabla {n}_{1,\delta }+{R}_{1}\left[\gamma ,\delta ,\alpha +\beta \right]{m}_{1,\beta }{n}_{1,\gamma }{n}_{1,\delta }=0$$

11 $${\eta }^{\beta }\frac{\partial {f}_{1,\beta }}{\partial t}+{\eta }^{\beta +\gamma }{m}_{1,\beta }\frac{\partial {n}_{1,\gamma }}{\partial t}+\frac{1}{2}{\eta }^{\beta +\gamma }\nabla {f}_{1,\beta }\nabla {f}_{1,\gamma }+\frac{1}{2}{S}_{1}\left[\beta ,\gamma ,0\right]{f}_{1,\beta }{f}_{1,\gamma }+{\eta }^{\beta +\gamma +\delta }\nabla {f}_{1,\beta }{m}_{1,\gamma }\nabla {n}_{1,\delta }+{S}_{1}\left[\beta ,\delta ,\gamma \right]{f}_{1,\beta }{m}_{1,\gamma }{n}_{1,\delta }+\frac{1}{2}{\eta }^{\beta +\gamma +\delta +\varepsilon }{m}_{1,\beta }{m}_{1,\gamma }\nabla {n}_{1,\delta }\nabla {n}_{1,\varepsilon }+\frac{1}{2}{S}_{1}\left[\delta ,\varepsilon ,\beta +\gamma \right]{m}_{1,\beta }{m}_{1,\gamma }{n}_{1,\delta }{n}_{1,\varepsilon }+g\eta +\frac{{p}_{1}}{{\rho }_{1}}=0$$

12 $${Q}_{1}\left[\alpha +\beta \right]=\frac{1}{\alpha +\beta +1}{\eta }^{\alpha +\beta +1}$$

13 $${R}_{1}\left[\alpha ,\beta ,\gamma \right]=\left\{\begin{array}{l}-\frac{\alpha \beta }{\alpha +\beta +\gamma -1}{\eta }^{\alpha +\beta +\gamma -1} \left(\alpha \beta \ne 0\right)\\ 0 \left(\alpha \beta =0\right)\end{array}\right.$$

14 $${S}_{1}\left[\alpha ,\beta ,\gamma \right]=\left\{\begin{array}{l}\alpha \beta {\eta }^{\alpha +\beta +\gamma -2} \left(\alpha \beta \ne 0\right)\\ 0 \left(\alpha \beta =0\right)\end{array}\right.$$

[2nd layer]

In the 2nd layer, i.e., the lower layer, $l=2$ and $j=1$, and the Euler–Lagrange equations become

15 $$-{\eta }^{\alpha }\frac{\partial \eta }{\partial t}+\nabla \left({Q}_{2}\left[\alpha +\beta \right]\nabla {f}_{2,\beta }\right)-{R}_{2}\left[\alpha ,\beta ,0\right]{f}_{2,\beta }+\nabla \left({Q}_{2}\left[\alpha +\beta +\gamma \right]{m}_{2,\beta }\nabla {n}_{2,\gamma }\right)-{R}_{2}\left[\alpha ,\gamma ,\beta \right]{m}_{2,\beta }{m}_{2,\gamma }=0$$

16 $${Q}_{2}\left[\alpha +\beta \right]\frac{\partial {m}_{2,\beta }}{\partial t}-{\eta }^{\alpha +\beta }{m}_{2,\beta }\frac{\partial \eta }{\partial t}+\nabla \left({Q}_{2}\left[\alpha +\beta +\gamma \right]\nabla {f}_{2,\beta }{m}_{2,\gamma }\right)-{R}_{2}\left[\alpha ,\gamma ,\beta \right]{f}_{2,\beta }{m}_{2,\gamma }+\nabla \left({Q}_{2}\left[\alpha +\beta +\gamma +\delta \right]{m}_{2,\beta }{m}_{2,\gamma }\nabla {n}_{2,\delta }\right)-{R}_{2}\left[\alpha ,\gamma ,\beta +\delta \right]{m}_{2,\beta }{m}_{2,\gamma }{n}_{2,\delta }=0$$

17 $${Q}_{2}\left[\alpha +\beta \right]\frac{\partial {n}_{2,\beta }}{\partial t}+{Q}_{2}\left[\alpha +\beta +\gamma \right]\nabla {f}_{2,\beta }{\nabla n}_{2,\gamma }+{R}_{2}\left[\beta ,\gamma ,\alpha \right]{f}_{2,\beta }{n}_{2,\gamma }+{Q}_{2}\left[\alpha +\beta +\gamma +\delta \right]{m}_{2,\beta }\nabla {n}_{2,\gamma }\nabla {n}_{2,\delta }+{R}_{2}\left[\gamma ,\delta ,\alpha +\beta \right]{m}_{2,\beta }{n}_{2,\gamma }{n}_{2,\delta }=0$$

18 $${\eta }^{\beta }\frac{\partial {f}_{2,\beta }}{\partial t}+{\eta }^{\beta +\gamma }{m}_{2,\beta }\frac{\partial {n}_{2,\gamma }}{\partial t}+\frac{1}{2}{\eta }^{\beta +\gamma }\nabla {f}_{2,\beta }\nabla {f}_{2,\gamma }+\frac{1}{2}{S}_{2}\left[\beta ,\gamma ,0\right]{f}_{2,\beta }{f}_{2,\gamma }+{\eta }^{\beta +\gamma +\delta }\nabla {f}_{2,\beta }{m}_{2,\gamma }\nabla {n}_{2,\delta }+{S}_{2}\left[\beta ,\delta ,\gamma \right]{f}_{2,\beta }{m}_{2,\gamma }{n}_{2,\delta }+\frac{1}{2}{\eta }^{\beta +\gamma +\delta +\varepsilon }{m}_{2,\beta }{m}_{2,\gamma }\nabla {n}_{2,\delta }\nabla {n}_{2,\varepsilon }+\frac{1}{2}{S}_{2}\left[\delta ,\varepsilon ,\beta +\gamma \right]{m}_{2,\beta }{m}_{2,\gamma }{n}_{2,\delta }{n}_{2,\varepsilon }+g\eta +\frac{{p}_{1}+\left({\rho }_{2}-{\rho }_{1}\right)g{h}_{1}}{{\rho }_{2}}=0$$

19 $${Q}_{2}\left[\alpha +\beta \right]=\frac{1}{\alpha +\beta +1}\left({\eta }^{\alpha +\beta +1}-{b}^{\alpha +\beta +1}\right)$$

20 $${R}_{2}\left[\alpha ,\beta ,\gamma \right]=\left\{\begin{array}{l}-\frac{\alpha \beta }{\alpha +\beta +\gamma -1}\left({\eta }^{\alpha +\beta +\gamma -1}-{b}^{\alpha +\beta +\gamma -1}\right) \left(\alpha \beta \ne 0\right)\\ 0 \left(\alpha \beta =0\right)\end{array}\right.$$

21 $${S}_{2}\left[\alpha ,\beta ,\gamma \right]=\left\{\begin{array}{l}\alpha \beta {\eta }^{\alpha +\beta +\gamma -2} \left(\alpha \beta \ne 0\right)\\ 0 \left(\alpha \beta =0\right)\end{array}\right.$$

where $j=0$ and 1, $\alpha$ = 0, 1, 2, …, $N-1$, $\beta$ = 0, 1, 2, …, $N-1$, $\gamma$ = 0, 1, 2, …, $N-1$, $\delta$ = 0, 1, 2, …, $N-1$, $\varepsilon$ = 0, 1, 2, …, $N-1$, and *b* is an arbitrary bottom (Fig. 1).

(8)–(21) can provide surface waves with vorticity effects over an arbitrary bottom in a two-layer fluid. By following Nakayama and Kakinuma^30^ and Nakayama and Lamb^35^, an implicit technique was used in the proposed numerical computational scheme in the one-dimensional region of the two-layer system in order to obtain stable computational results.

For the numerical analysis, the depths of the upper and lower layers were ${h}_{1}=4.0$ m and ${h}_{2}=2.0$ m, the specific density ratio between the upper and lower layers was ${\rho }_{1}/{\rho }_{2}=1/1000$, and the computational domain size was 250.0 m, with 500 grids and a time step of 0.00025 s, which corresponds to about CFL 1/400. Three wave amplitudes were given as initial conditions: 0.20 m, 0.35 m, and 0.70 m (Table 1). The term of the vertically distributed function was given as $N=2$ by following a previous study regarding solitary waves^34^. Five vertically uniform vorticities were given: ${\omega }_{0}=0.0$ s^−1^, ${\omega }_{0}=0.25$ s^−1^, ${\omega }_{0}=0.50$ s^−1^, ${\omega }_{0}=-0.25$ s^−1^, and ${\omega }_{0}=-0.50$ s^−1^. Radiation conditions were applied at the left and right boundaries. As initial conditions, the 3rd-order solution based on Mirie and Pennell^42^ and Nakayama et al.^33^ was used to target large-amplitude solitary waves. Note that we applied the initial conditions of Mirie and Pennell^42^ and Nakayama et al.^33^ here because of the necessity of giving velocity potentials in both the upper and lower layers as initial conditions for the FDV-2s equations.

Since we considered surface waves, the vorticity effect was given only in the lower water layer. In the analysis, vertically uniform vorticity was given as ${\omega }_{0}$ (s^−1^). Vorticity, $\omega$, can be expressed by using the additional terms of velocity indicated by µ_*l*_ and ${\nu }_{l}$ of (1) as (22).

22 $$\omega =\frac{\partial w}{\partial x}-\frac{\partial u}{\partial z}=\frac{\partial {\mu }_{l}}{\partial x}\frac{\partial {\nu }_{l}}{\partial z}-\frac{\partial {\nu }_{l}}{\partial x}\frac{\partial {\mu }_{l}}{\partial z}$$

It is necessary to include the second term of vertically distributed functions for ${\mu }_{2}$ and ${\nu }_{2}$ in order to give a vertically uniform vorticity, ${\omega }_{0}$ s^−1^, which yields (23).

23 $${\omega }_{0}={n}_{\mathrm{2,1}}\frac{\partial }{\partial x}\left({m}_{\mathrm{2,0}}+{m}_{\mathrm{2,1}}z\right)-{m}_{\mathrm{2,1}}\frac{\partial }{\partial x}\left({n}_{\mathrm{2,0}}+{n}_{\mathrm{2,1}}z\right)$$

To give horizontally constant values, ${n}_{\mathrm{2,1}}=0$ yields ${n}_{\mathrm{2,0}}$ as follows:

24 $${n}_{\mathrm{2,0}}=-\frac{{\omega }_{0}}{{m}_{\mathrm{2,1}}}x$$

To obtain coefficients ${m}_{\mathrm{2,0}}$ and ${m}_{\mathrm{2,1}}$, it is assumed that the vertically integrated horizontal velocity due to the vorticity effect is zero, which gives (25). Since the coefficient ${m}_{2,0}$ can be taken as any constant value, we can give ${m}_{\mathrm{2,0}}=1$.

25 $${m}_{\mathrm{2,1}}=\frac{2}{2{h}_{1}+{h}_{2}}$$

## Conclusion

Laboratory experiments were conducted successfully by using roughness at the open channel bottom to investigate the effect of negative vorticity when a solitary wave progresses in the positive direction. The Froude number was − 0.0077, but the decrease in wave height around the trough was confirmed to be similar to the decreases in previous studies by Choi^11^ and Teles and Peregrine^9^. The Nakayama model was used to investigate the influence of the Froude number on a solitary wave. Vertically uniform and constant vorticity was applied to progressive solitary waves. When positive vorticity was given, the effective wavelength increased compared to the no-vorticity case. Also, wave height decreased with positive vorticity, resulting in the generation of a wave with more extended wavelength, which has more significant normalized total energy than a wave with no vorticity (Fig. 4).

## Data Availability

The data that support the findings of this study are available from the corresponding author upon reasonable request.
